# Enhancing Speech Recognition Using Improved Particle Swarm Optimization Based Hidden Markov Model

**DOI:** 10.1155/2014/270576

**Published:** 2014-11-17

**Authors:** Lokesh Selvaraj, Balakrishnan Ganesan

**Affiliations:** ^1^Department of Computer Science & Engineering, Hindusthan Institute of Technology, Coimbatore, Tamil Nadu 641 032, India; ^2^Indra Ganesan College of Engineering, Trichy, Tamil Nadu 620 012, India

## Abstract

Enhancing speech recognition is the primary intention of this work. In this paper a novel speech recognition method based on vector quantization and improved particle swarm optimization (IPSO) is suggested. The suggested methodology contains four stages, namely, (i) denoising, (ii) feature mining (iii), vector quantization, and (iv) IPSO based hidden Markov model (HMM) technique (IP-HMM). At first, the speech signals are denoised using median filter. Next, characteristics such as peak, pitch spectrum, Mel frequency Cepstral coefficients (MFCC), mean, standard deviation, and minimum and maximum of the signal are extorted from the denoised signal. Following that, to accomplish the training process, the extracted characteristics are given to genetic algorithm based codebook generation in vector quantization. The initial populations are created by selecting random code vectors from the training set for the codebooks for the genetic algorithm process and IP-HMM helps in doing the recognition. At this point the creativeness will be done in terms of one of the genetic operation crossovers. The proposed speech recognition technique offers 97.14% accuracy.

## 1. Introduction

Speech recognition is also known as automatic speech recognition (ASR) which may provide an accurate identification of authorized speech signals. Many applications are developed based on ASR such as text to speech system, public address system, mobile, and personal communication [1, 2]. Some of the ASR systems may not recognize accurate speech signals, because they may suffer from any of these challenges such as denoising speech signals, extraction of appropriate feature vector, feature selection, and recognition methods [3]. Generally appropriate feature selection and proper recognition [4, 5] are very important to increase accuracy in speech recognition so proposed work mainly emphasizes these areas.

The proposed speech recognition technique is based on improved particle swarm optimization based hidden Markov model (IP-HMM) technique which uses the concept of Mel frequency cepstral coefficients (MFCC) for determining feature extraction. MFCC is one of the most thriving feature representations in speech recognition connected tasks, and the coefficients are acquired through a filter bank study. The measures involved in the features extraction are preemphasis, frame blocking, windowing, filter bank analysis, logarithmic compression, and discrete cosine transformation [6]. For feature selection, genetic vector quantization (VQ) based algorithm is used for mapping vectors (feature extraction of input signal) from a large vector space to a finite number of regions in that space. Each region is called a cluster and can be represented by its center called a code word. The collection of all code words is called a codebook [7, 8].

IP-HMM helps for speech recognition which is achieved by competently finding optimal or near optimal solutions in large search spaces of code vector from codebook. There are two dissimilar kinds of versions that are employed according to IPSO [9]: (i) “individual best” (*pbest*) and (ii) “global best” (*gbest*). The working mechanism is involved in sequence of steps such as swarm initialization, computing the fitness function, *pbest* and *gbest* initialization, swarm update, and criteria to stop. During training and testing phase for speech input signals, if *gbest* and *pbest* values are appropriately the same then the speech signals are recognized accurately. Speech recognition accuracy is obtained by number of speech signals recognized out of total number of signals substituted during testing.

The proposed technique is trained with 35 speech signals collected from various speakers where each speech signal contains a word. Totally 10 speakers were used in training process, among them five males and five females. The effect of IP-HMM is tested with 165 speech signals collected from 13 speakers out of which seven were males and six were females. Tested signal contains combination of isolated words (elephant, vegetables, etc.) and connected words (operating system, USA, etc.). The performances of this work were analysed based on speech recognition accuracy with the three existing speech recognition systems such as speech recognition using neural network [10, 11], hidden Markov model [12, 13], and particle swarm optimization [14]. As a result, improved particle swarm optimization technique is employed offering more accurate result.

## 2. Materials and Methods Relinquish

### 2.1. Proposed Methodology

**Figure 1:**
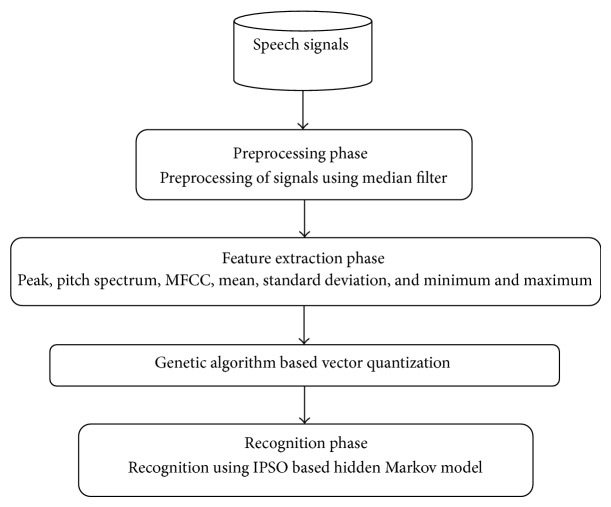
Architecture of our proposed methodology.

The main objective of this research is to enhance speech recognition. In the proposed method, a novel enhancing speech recognition method is based on vector quantization and improved particle swarm optimization (IPSO) is suggested. The suggested methodology contains four stages, namely, (i) denoising, (ii) feature mining, (iii) vector quantization, and (iv) IPSO based HMM technique called IP-HMM.  Figure 1 illustrates the architecture of proposed method, which was involved in sequence of process. Initially the speech signals are collected as input from the speakers and it is denoised with aid of median filter in preprocessing phase. In the next step, attributes such as peak, pitch spectrum, MFCC, mean, standard deviation, and minimum and maximum of the signal are extorted from the denoised signal. Following that, to reach the training process, the extorted features are prearranged to genetic algorithm based codebook generation in vector quantization. The codebook is trained with thirty-five speech signals and is next tested by applying 165 speech signals. The codebooks for the genetic algorithm process, initial population, are formed by choosing random code vectors from the training set. The concert of the suggested method is examined by giving more speech signals to the guided codebook in noisy and clean environments. At last, the recognition will be made by improved particle swarm optimization based hidden Markov model. At this point the improvisation will be made in terms of one of the genetic operation crossovers.

#### 2.1.1. Preprocessing

The median filter is a nonlinear digital filtering method, which is employed to eradicate noise. For developing the results of later processing steps, this noise reduction is a preprocessing step. Median filters are nonlinear rank-order filters based on substituting each element of the source vector with the median value, taking over the fixed neighbourhood of the processed element. These filters are widely applied in signal processing applications. While keeping the signal blurring to the minimum, median filtering is used to remove the spontaneous noise in the signal. The most important scheme of the median filter is analysing the signal based on the entries and replacing each entry with the median of neighbouring entries.

#### 2.1.2. Feature Extraction

In order to attain the desired speech processing tasks, the exact features are removed from the input noise-free speech signals at this point. In order to give up an enhanced recognition performance, the removal of the best parametric representation of acoustic signals is an important task. The competence of this phase is crucial for the next phase. MFCC is one of the most thriving feature representations in speech recognition connected tasks, and the coefficients are acquired through a filter bank study. The characteristics such as peak, pitch spectrum, MFCC, mean and standard deviation of the signal, and minimum and maximum of the signal are extorted from the denoised signal. 

*(i) Peak (P).* Peak is the maximum level in a signal. The peak is extorted by means of the MATLAB function called “Peak Finder.” In the stepwise computation of peak finding technique undergoes from the problem that if the signal encloses noise, fake signals are furthermore identified as peaks. But this function goes after a dissimilar nature of derivate besides with the user defined threshold to locate the local maxima or minima in peak recognition. Using a user defined magnitude threshold, this function discovers local peaks or valleys (local maxima) in a noisy vector to find out if each peak is considerably larger or smaller than the data around it. 

*(ii) Pitch Spectrum (PS)*. Pitch is the smallest frequency component of a signal that inspires to a vocal system. Pitch period is the least repeating signal which is indirectly relative to the fundamental frequency. To demonstrate the pitch signal totally, pitch period is employed. The YAAPT (yet another algorithm for pitch tracking) is a basic frequency (pitch) tracking algorithm, which is designed to be extremely precise and very vigorous for both high quality and telephone speech [15]. The YAAPT algorithm contains five steps.

(1) Preprocessing: two versions of signals, that is, original signal and absolute value of the signal, are generated and each signal is band pass filtered and center clipped.
(2) Pitch candidate selection based on normalized cross correlation function (NCCF): the association signal has a peak of huge magnitude at a delay parallel to the pitch period. If the magnitude of the largest peak is higher than threshold (about 0.6), then the frame of speech is voiced frequently.
(3) To find candidate refinement based on spectral information, the candidate attained in the earlier step is adapted based on the global and local spectral information.
(4) Candidate modifications based on plausibility and continuity constraints: a soft pitch track is attained by adapting the purified candidate with the assist of normalized low frequency energy ratio (NLFER) by using (1). NLFER is computed to help indicate voiced and unvoiced regions in the speech signal [16]. The sum of absolute values of spectral samples over the low frequency regions is taken and then normalized by dividing by the average low frequency energy per frame over the speech signal. Consider
  (1) $$\begin{array}{c} \text{NLFER}=\frac{\sum_{i}^{}x\left(i,j\right)}{\left(1/N\right)\sum_{i}^{}\sum_{j}^{}x\left(i,j\right)}, \end{array}$$
 where *N* is total number of frames, *i* is frequency index, *x* (*i*, *j*) is log magnitude of low frequency region, and *j* is frame index.
(5) Finally path determination by means of dynamic programming: pitch candidate matrix, a merit matrix, an NLFER curve (from the original signal), and the spectrographic pitch track attained in the above steps are used to discover the lowest cost pitch track among all accessible candidates by means of dynamic programming.

*(iii) Minimum and Maximum of the Signal.* The highest value of the signal is called maximum of the signal (max) and the lowest value in the signal is called minimum of the signal (min). These computed characteristics are then fed as key in to the genetic vector quantization in order to produce the codebook.

#### 2.1.3. Genetic Algorithm and Vector Quantization

The vector quantization (VQ) approach is used for mapping vectors (feature extraction of input signal) from a large vector space to a finite number of regions in that space. Each region is called a cluster and can be represented by its center called a code word. The collection of all code words is called a codebook. Here the codebook is based on entire words and the method of vector quantization contains extorting a small number of representative characteristic vectors as a competent means of characterizing the speaker exact features. Using vector quantization, storing every single vector that is produced from the training is not possible. In the training phase, using the clustering algorithm explained in a vector quantization codebook is produced for each clustering of the training vectors. The distance from a vector to the adjoining code word of a codebook is called a VQ-distortion. In our strategy, a genetic vector quantization based algorithm is improving for signal compression, and the algorithm to condense signal is demonstrated as in the following.

(1) Find out the number of code words, *N*, or the size of the codebook.
(2) Choose *N* code words at random, and let that be the first codebook. The first code words can be arbitrarily selected from the set of input vectors.
(3) Using the Euclidean distance, measure the clustered vectors around each code word. This is made by taking each input vector and finding the Euclidean distance among each code word. The input vector belongs to the cluster of the code words that gives up the minimum distance.
(4) Calculate the novel set of code words. This is made by acquiring the average of each cluster. Include the component of each vector and separate by the number of vectors in the cluster. It is given by
  (2) $$\begin{array}{c} y_{i}=\frac{1}{m}\overset{m}{\underset{j=1}{\sum }}x_{ij}, \end{array}$$
 where *i* is the component of each vector, *m* is the number of vectors in the cluster,  and *x*_*ij*_ is the vector component.
(5) Do Steps (2) and (3) again till the code words do not differ or the changes in the code words are little.

**Figure 2:**
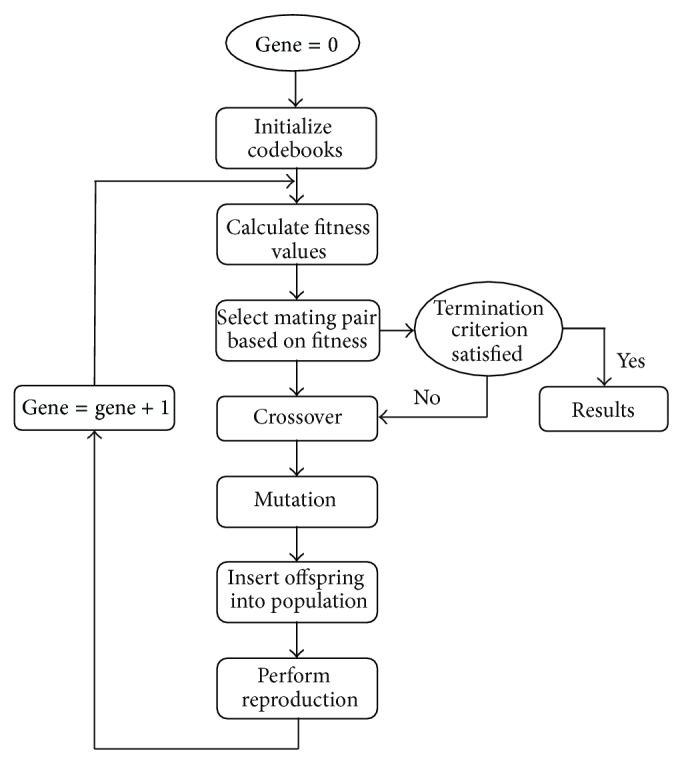
Sequence of steps involved in genetic algorithm based vector quantization.

After codebook generation genetic algorithm is implemented in order to get optimal codebook. The actual genetic algorithms can be employed on a qualified searching mechanism with the Darwinian thoughts of the continuing existence of the fittest along with interbreeding regarding healthy parents. Genetic algorithm functions in the more stochastic means in comparison with conventional search algorithm. To discover optimal solution, carefully guided randomness enables the genetic algorithm much quicker in comparison with the deterministic counterparts. The actual encouraged strategy is usually to discover ideal codebook with a genetic algorithm which in turn engages a regular structure for the action, which in turn consists of fitness, selection, crossover, and mutation along with ceasing conditions. Particularly the number of training vectors *M* along with the number of code vectors *N*, the codebook pattern is usually to categorize the *M* coaching vectors into *N* groups. The pseudocode for genetic algorithm is given (Pseudocode 1) and Figure 2 shows the sequence of steps involved in genetic algorithm based vector quantization for genetic initialization, selection with fitness computation, crossover, mutation, and termination criteria.

**Step 1 (genetic initialization).**

Each chromosome stands for a codebook. The label *I* (*I* = 1,2, 3,…………, *M*) of the training vector is analysed as a gene; as a result the fundamental individual is made up of *M* genes which can divide into *N* units, and each unit is made up of numerous labels which belong to this unit. *N* code words are the centre vector of the training vectors in each unit. The information structure of codebook will be an array of two dimensions. The number of rows will be 256∗16 and this signifies the length of code word, whereas the number of columns in the codebook will fluctuate according to the input signal.

**Step 2 (selection).**

Choose two parents for crossover and to produce novel offspring(s). In this technique, two dissimilar individuals are chosen arbitrarily. The individual that has higher fitness will succeed to be added to the crossover mate. If the fitness value of the first individual is identical to the fitness value of the second individual, one of them will be chosen arbitrarily. The fitness is computed based on the input vector and the codebook vector by

(3) $$\begin{array}{c} f_{i}=\frac{1}{\left(1/N\right)\sum_{j=1}^{n}\sum_{i=1}^{k}\left(X_{j}\;-\;Y_{i}\right)}, \end{array}$$

where *f*_*i*_ is fitness value,  *N* is number of signals, *X*_*j*_ are values of the speech signals, *Y*_*i*_ are values of the code vector.

**Step 3 (crossover).**

Crossover is the method of exchanging the parents' genes to generate one or two offsprings that transmit inherent genes from both parents. Now two point crossovers are applied. It works by picking two arbitrary points inside two parent chromosomes, and then exchanging the genes among these points in each parent to generate two novel offspring. This is executed with a certain possibility; or else the two parents are copied as offsprings.

**Step 4 (mutation).**

In genetic algorithm, mutation is the concluding stage. Mutation is a genetic operator which is applied to uphold genetic diversity from one generation of a population of algorithm chromosomes to the next. To uphold genetic diversity, a genetic operator is an operator employed in genetic algorithms. In mutation the chromosome value stays the same.

**Step 5 (termination criteria).**

We employed number of generations in the suggested algorithm and error as stopping criteria. When one of the two conditions is pleased the program will discontinue.

#### 2.1.4. Improved Particle Swarm Optimization Based Hidden Markov Model

To prove that IPSO is potentially useful for evolving HMMs, we implemented a standard IPSO process where a population of HMMs is evolved.

Population based search algorithm is known to be particle swarm optimization (PSO). It is formed to pretend the manners of birds in hunt for food on a cornfield or fish school. The technique can competently find optimal or near optimal solutions in large search spaces. There are two dissimilar kinds of versions which are employed according to PSO: (i) “individual best” and (ii) “global best.”

*(i) “Individual Best.”* It is the individual best selection algorithm by assessing each individual position of the particle to its own best position *pbest*(*pb*), only. The information about the other particles is not used in this *pbest*.

*(ii) “Global Best.”* It is the universal best selection algorithm *gbest*(*gb*), which attains the global information by making the movement of the particles enclose the position of the best particle from the whole swarm. Besides, every particle uses its experience with earlier incidents in terms of its own best solution. Consider

(4) $$\begin{array}{c} V_{i}^{d}=V_{i}^{d}+c_{1}\cdot r_{1}\cdot \left(pb_{i}^{d}-x_{i}^{d}\right)+c_{2}\cdot r_{2}\cdot \left(gb^{d}-x_{i}^{d}\right)\\ x_{i}^{d}=x_{i}^{d}+\delta V_{i}^{d}. \end{array}$$

Each individual particle *i* has a randomly initialized position *X*_*i*_ = (*x*_*i*_^1^, *x*_*i*_^2^,…, *x*_*i*_^*D*^), where *x*_*i*_^*d*^ is its position in the *d*th dimension, velocity is *V*_*i*_ = (*v*_*i*_^1^, *v*_*i*_^2^,…, *v*_*i*_^*D*^), where *v*_*i*_^*d*^ is the velocity in the *d*th dimension, *pb*_*i*_ = (*pb*_*i*_^1^, *pb*_*i*_^2^,…, *pb*_*i*_^*D*^), where *pb*_*i*_^*d*^ is the best position in the *d*th dimension, and *gb* = (*gb* ^1^, *gb* ^2^,…, *gb* ^*D*^), where *gb* ^*d*^ is the global best position in the *d*th dimension in the D-dimensional search space. Every particle can go in the direction of its personal best position to its best global position in the course of each generation. The mixing process of a swarm particle in the search space is described as in (4):

 
*c*_1_, *c*_2_: constants with the value of 2.0,
 
*r*_1_, *r*_2_: independent random numbers generated in the range [0-1],
 
*V*_*i*_^*d*^: velocity of *i*th particle,
 
*x*_*i*_^*d*^: current position of the particle *i*,
 
*pb*_*i*_^*d*^: best fitness value of the particle at the current iteration,
 
*gb* ^*d*^: best fitness value in the swarm.

*Disadvantages of PSO.* The search direction is not obvious and offers slow convergence. There is no utilization by presenting extra information as it did not take the benefit of the additional information.

As a result, improved particle swarm optimization (IPSO) technique is employed offering more accurate result. Now, the peak, pitch spectrum, optimised codebook, and MFCC were optimized by means of IPSO.

#### 2.1.5. MFCC, Peak, Pitch Spectrum, and Codebook Parameter Optimization by IPSO

**Figure 3:**
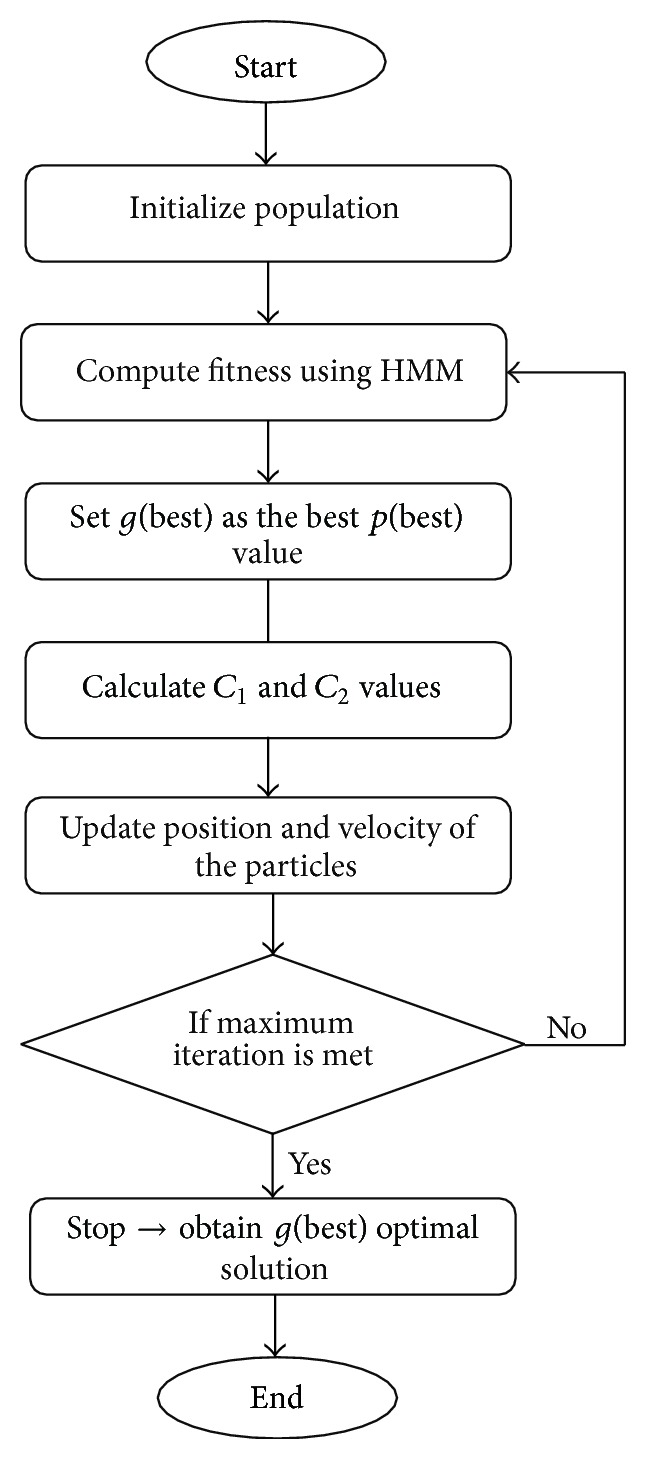
Working Mechanism of IPSO.

The working mechanism of the IPSO is shown in Figure 3 and it is explained below.

(i) *Swarm Initialization*: for a population size *u*, produce the particles arbitrarily.
(ii) *Define the Fitness Function*: according to the present population, the fitness function selected should be applied for the constraints. The fitness function is given in
  (5) $$\begin{array}{c} \text{Sequence}=\text{hmm}\;\text{generate}\;\left(2,\text{inp},\text{tar}\right)\\ \text{Fitness}=\text{hmmtrain}\;\left(\text{Seq},\text{inp},\text{tar}\right). \end{array}$$
(iii) *pb* *and* *gb* *Initialization*: at first the fitness value computed for each particle is set as the *Pbest* value of each particle. Among the *Pbest* values, the best one is chosen as the *gb* value.
(iv) *C*_1_ and *C*_2_ values are calculated by using (6).
 Assume that *C*_max⁡_ = 3 and *C*_min⁡_ = 1:
  (6) C1=Cmax⁡Cmin⁡∗Cmin⁡fitMeanfit+  Min⁡fit2∗max⁡fit +  Cmin⁡C2  =  C1.
(v) *Velocity Computation*: the novel velocity is computed by means of
  (7) $$\begin{array}{c} V_{i}^{d+1}=V_{i}^{d}+c_{1}\cdot r_{1}\cdot \left(pb_{i}^{d}-x_{i}^{d}\right)+c_{2}\cdot r_{2}\cdot \left(gb^{d}-x_{i}^{d}\right) \end{array}$$
  (8) $$\begin{array}{c} x_{i}^{d}=x_{i}^{d}+\delta V_{i}^{d}. \end{array}$$
(vi) * Swarm Update*: work out the fitness function once more and amend the *pb* and *gb* values. If the new value is better than the earlier one, replace with the old by the current one. And moreover select the best *pb* as the *gb*.
(vii) * Criterion to Stop*:  sustain until the solution is good enough or maximum iteration is accomplished.

Pseudocode for IPSO based hidden Markov model is given (Pseudocode 2) where the hmm functions were used as fitness for the IPSO algorithm. Then the best fitness *pbest* and the *gbest* which represent the best among the *pbest* values were identified. The particle position and velocity values were calculated. In ordinary PSO the *C*_1_ and *C*_2_ were considered as constants and in IPSO the *C*_1_ and *C*_2_ values were calculated using the corresponding equation mentioned in (6). When the maximum iterations were met the process stopped.

## 3. Experiments

The proposed speech recognition technique is implemented in the working platform of MATLAB (version 7.12). It is trained using 35 speech signals collected from various speakers where each speech signal of a speaker contains a word as shown in Table 1. Totally 10 speakers were used among them, five male and five female speakers.

Later experiment is tested with 165 speech signals collected from 13 speakers out of which seven were males and six were females. Among 165 speech signals few are listed in Table 2; the tested signal contains combination of isolated and connected words.

**Figure 4:**
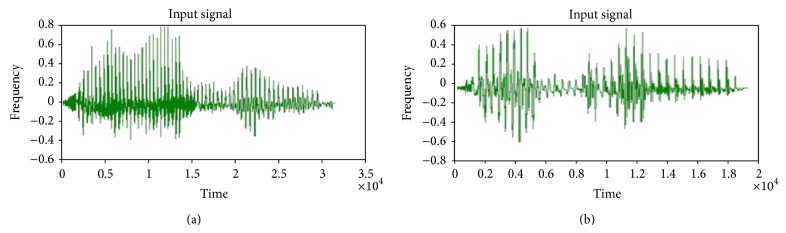
Input speech signals contain words (a) speech recognition and (b) kingfisher.

For experimental purpose, four input speech signals (dog, speech recognition, kingfisher, and jasmine) were employed here and two input signals among them are shown in Figure 4. The input signals in Figures 4(a) and 4(b) represent the words containing “speech recognition” and “kingfisher.”

**Figure 5:**
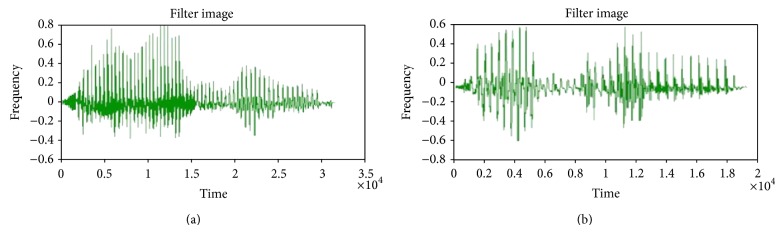
Median filter output for the two input signals (a) and (b), respectively.

Input speech signals are gathered for our work and then these signals are subjected to preprocessing step by means of median filter. The speech signals that get preprocessed and each signal are median filtered separately and this output is obtained. The median filter output for the given signals illustrated in the subsequent Figures 5(a) and 5(b) represents the words “speech recognition” and “kingfisher,” respectively.

**Figure 6:**
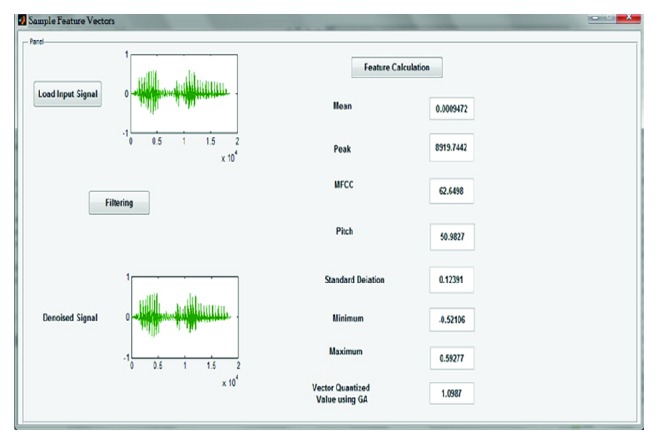
Screenshot of feature extraction of input signal (kingfisher).

Next, median filter output signals are used to find the feature extraction such as peak, pitch spectrum, MFCC, mean, standard deviation, and minimum and maximum of the signal.  Figure 6 shows a sample screenshot of feature extraction of a signal (kingfisher).

**Figure 7:**
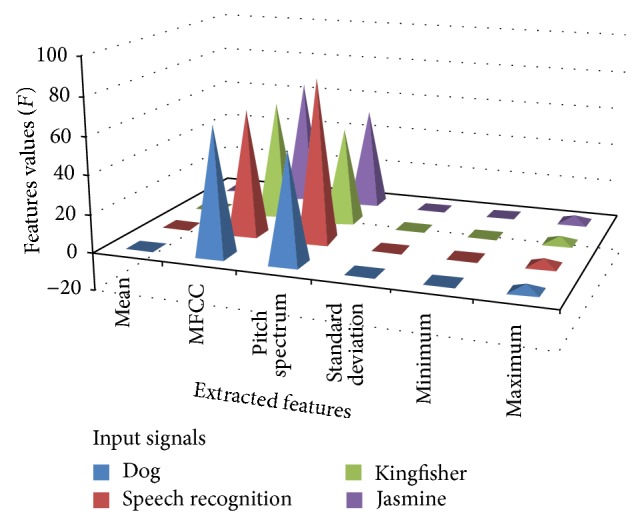
Plots for extracted features of input signals.

The outcomes of various features extraction of four input signals were given in Table 3 and the graphical representation of the extracted features is given in Figure 7 except the peak values of the input signal.

**Figure 8:**
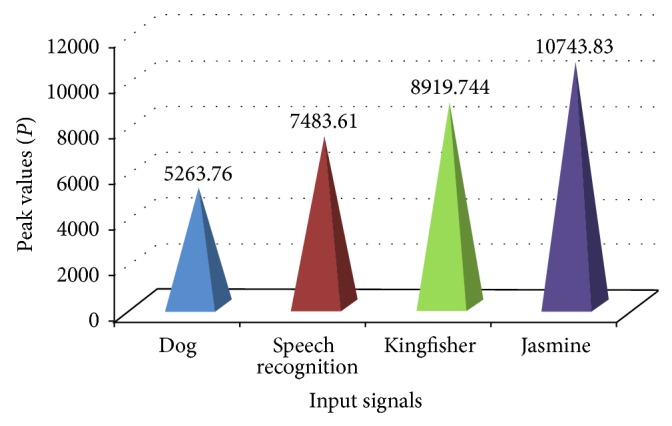
Peak value of the input signals.

The maximum level in a signal is called peak. The peak values of the four input signals are graphically represented in Figure 8.

**Figure 9:**
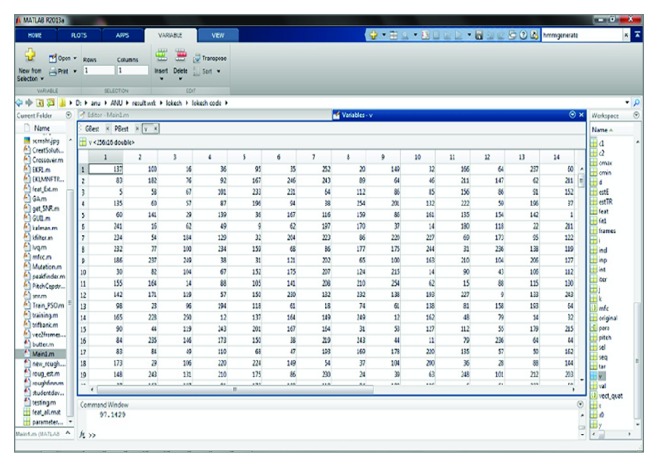
Screenshot for vector quantization values.

**Figure 10:**
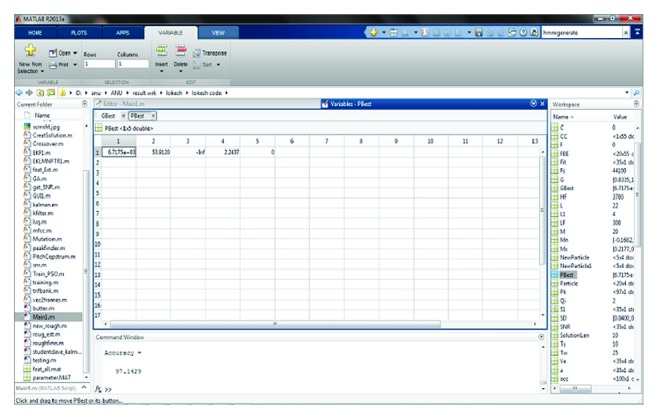
Screenshot for the *gbest* values calculation.

**Figure 11:**
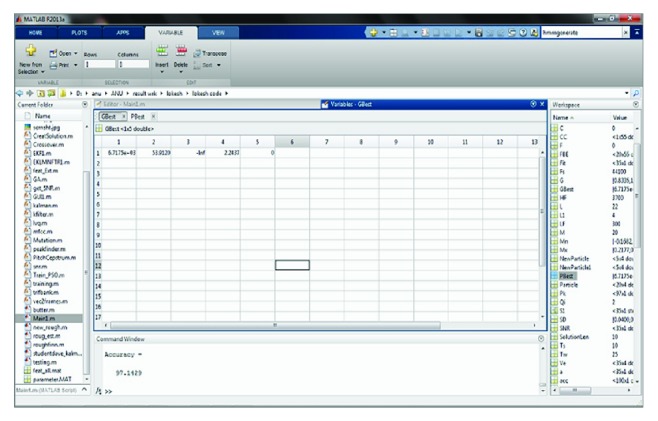
Screenshot for the  *pbest* values calculation.

Following that, to accomplish the training process, the extracted feature values are given to genetic algorithm based codebook generation in vector quantization. The obtained vector quantization values of all the input signals used in training set are shown in Figure 9. The initial populations are created by selecting random code vectors from the training set for the codebooks for the genetic algorithm process and by computing pseudocode for IPSO based hidden Markov model (IP-HMM) in Section 2.1.5. During training and testing phase for speech input signals, if *gbest* and *pbest* values are appropriately the same then the speech signals are recognized accurately. Figures 10 and 11 show the screenshot of obtained *gbest* and *pbest*  value for the input signals. At this point the creativeness will be done in terms of one of the genetic operation crossovers.

Our suggested work recognizes whether the speech signal is familiar or unfamiliar. The overall speech recognition accuracy value is obtained by means of IPSO is given in (9). Speech recognition accuracy is measured in terms of number of speech signals recognized out of total number of signals substituted during testing:

(9) $$\begin{array}{c} \text{Speech}\;\text{Recognition}\;\text{Accuracy}\;=\left(\frac{\text{NS}}{\text{TNS}}\right)\times 100, \end{array}$$

where NS is number of speech signals recognized and TNS is total number of speech signals substituted.

The performances of the proposed work were analysed with three speech recognition systems such as speech recognition using neural network, hidden Markov model, and particle swarm optimization in terms of speech recognition accuracy obtained during testing. As a result, improved particle swarm optimization (IPSO) technique is employed offering more accurate result.

## 4. Results and Discussion

Result of proposed work is evaluated based on number of speech signals recognized attain during testing. Totally 13 speakers were used for testing and each speaker is tested with 165 speech signals and it is obtained that, for thirteen speakers, 14587 speech signals were recognized out of 15105 speech signals. The proposed speech recognition technique using IPSO based HMM offers 97.14% of speech recognition accuracy as in (10) and it is compared with NN, HMM, and PSO by using same speech signals of the speakers as given in Table 4. The experimental result shows that the method NN attains 88.58%, HMM attains 78.33%, and PSO attains 84.34% of speech recognition accuracy. This reveals that proposed work improves the speech recognition accuracy by 8.56% compared with NN, 18.81% with HMM, and 12.80% with PSO. Consider

(10) $$\begin{array}{c} \text{Speech}\;\text{Recognition}\;\text{Accuracy}=\left(\frac{14587}{15105}\right)\times 100=97.14\%. \end{array}$$

**Figure 12:**
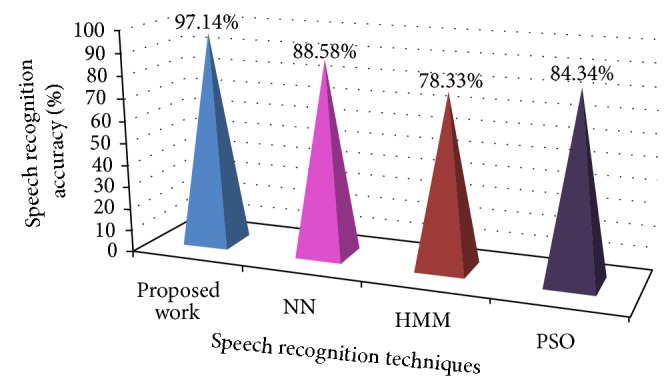
Perfomance comparison of proposed work with NN, HMM, and PSO.

In all cases, evaluate the values to the NN, PSO, and HMM technique and it is discovered that the suggested method executes superiorly in terms of accuracy as shown in Figure 12.

## 5. Conclusion

The primary intention of this research is speech recognition with the aid of IPSO based HMM. Our suggested work recognizes whether the speech signal is familiar or unfamiliar. The overall speech recognition accuracy value obtained by this experiment is 97.14%. The performance is compared with three speech recognition techniques, NN, HMM, and PSO. The comparison result shows that proposed work improves the speech recognition accuracy by 8.56% compared with NN, 18.81% with HMM, and 12.80% with PSO. Therefore by utilizing the IPSO based HMM, the proposed speech recognition technique proficiently recognized the speech signal.

## Figures and Tables

**Pseudocode 1:**
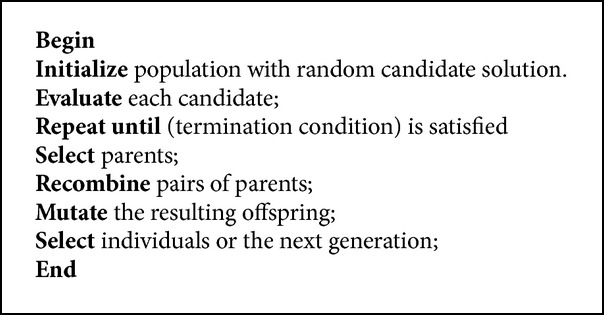
Pseudocode for genetic algorithm.

**Pseudocode 2:**
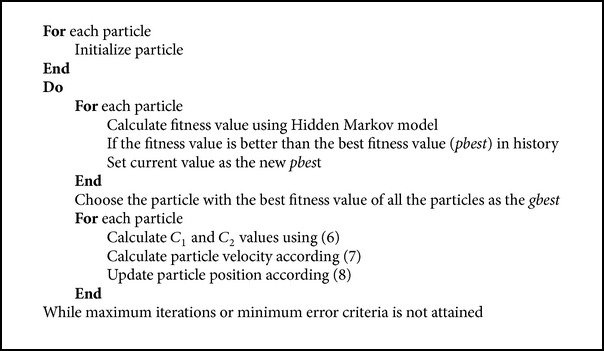
Pseudocode for IPSO based hidden Markov model (IP-HMM).

**Table 1:** Speech signals used for training.

35 speech signals					
Dog	Peacock	Dinosaurs	Fish	Moon	Uniform
Eyebrow	Rose	Dove	Flower	Nest	Vegetables
Jasmine	Sunflower	Duck	Fruits	Orange	Village
Kingfisher	Tiger	Eagle	Joker	Queue	Wild animals
Marine	Buffalo	Elephant	Kingfisher	Rabbit	welcome
Lotus	Cow	Eagle	Kitten	Six	Zebra

**Table 2:** Sample speech signals used for testing.

Few speech signals					
Computer	Water bottle	Season	Dictionary	Electronics	Technology
Software	Mouse	Raining	Commercial	Mechanical	Ocean
Hardware	Blossom	Towel	Intermediate	Medicine	Laptop
Operating system	Control system	System software	Web applications	Speech recognition	Research Centre
Input device	Heterogeneous	Thousand	National	Polar beer	University
Output device	Software engineering	Hundred	Country	Penguin	United states

**Table 3:** Values of features extracted from the input signal.

Input signals	Features extracted from the signal						
	Mean	Peak	MFCC	Pitch spectrum	Standard deviation	Minimum	Maximum
Dog	0.001249	5263.76	67.3001	57.95	0.1184	−0.3915	0.74557
Speech recognition	0.001857	7483.61	66.222	85.6310	0.08955	−0.3334	0.82226
Kingfisher	0.000947	8919.744	62.64977	50.98265	0.123912	−0.521057	0.592773
Jasmine	0.001917	10743.83	65.72812	52.68817	0.102743	−0.459289	0.925898

**Table 4:** Performance comparison of proposed work with NN, HMM, and PSO.

Number of speakers	Total number of speech signals (TNS) tested	Number of speech signals recognized (NS)			
		Proposed work	NN	HMM	PSO
1 speaker	165	162	153	136	144
2 speakers	330	323	303	268	284
3 speakers	495	485	451	396	426
4 speakers	660	645	598	511	564
5 speakers	825	806	742	641	705
6 speakers	990	968	885	767	835
7 speakers	1155	1127	1029	880	978
8 speakers	1320	1286	1171	1001	1116
9 speakers	1485	1443	1307	1127	1256
10 speakers	1650	1599	1448	1245	1391
11 speakers	1815	1756	1598	1366	1520
12 speakers	1980	1916	1723	1486	1659
13 speakers	2145	2071	1893	1603	1787
Total	**15015**	**14587**	**13301**	**11427**	**12665**
Speech recognition accuracy		**97.14%**	**88.58%**	**78.33%**	**84.34%**
